# CD24 is a surrogate for ‘immune‐cold’ phenotype in aggressive large B‐cell lymphoma

**DOI:** 10.1002/cjp2.266

**Published:** 2022-03-14

**Authors:** Morihiro Higashi, Shuji Momose, Natsuko Takayanagi, Yuka Tanaka, Tomoe Anan, Takahisa Yamashita, Jun Kikuchi, Michihide Tokuhira, Masahiro Kizaki, Jun‐ichi Tamaru

**Affiliations:** ^1^ Department of Pathology, Saitama Medical Center Saitama Medical University Kawagoe Japan; ^2^ Department of Hematology, Saitama Medical Center Saitama Medical University Kawagoe Japan; ^3^ Hematology, Saitama Medical Center Japan Community Health Care Organization Kawagoe Japan

**Keywords:** lymphoma, DLBCL, tumor microenvironment

## Abstract

The tumor microenvironment (TME) is a critical regulator of the development of malignant lymphoma. Therapeutics targeting the TME, especially immune checkpoint molecules, are changing the treatment strategy for lymphoma. However, the overall response to these therapeutics for diffuse large B‐cell lymphoma (DLBCL) is modest and new targets of immunotherapy are needed. To find critical immune checkpoint molecules for DLBCL, we explored the prognostic impact of immune checkpoint molecules and their ligands using publicly available datasets of gene expression profiles. *In silico* analysis of three independent datasets (GSE117556, GSE10846, and GSE181063) revealed that DLBCL expressing CD24 had a poor prognosis and had a high frequency of *MYC* aberrations. Moreover, gene set enrichment analysis showed that the ‘MYC‐targets‐hallmark’ (false discovery rate [FDR] = 0.024) and ‘inflammatory‐response‐hallmark’ (FDR = 0.001) were enriched in CD24‐high and CD24‐low DLBCL, respectively. In addition, the expression of cell‐specific markers of various immune cells was higher in CD24‐low DLBCL than in CD24‐high DLBCL. CIBERSORT analysis of the datasets showed fewer macrophages in CD24‐high DLBCL than in CD24‐low DLBCL. Additionally, immunohistochemical analysis of 335 cases of DLBCL showed that few TME cells were found in CD24‐high DLBCL, although statistical differences were not observed. These data indicate that CD24 expression suppresses immune cell components of the TME in DLBCL, suggesting that CD24 may be a target for cancer immunotherapy in aggressive large B‐cell lymphoma.

## Introduction

The tumor microenvironment (TME), including the immune system, is a critical regulator of the development of malignant lymphoma. Chen and Mellman described the cancer–immunity cycle, a set of self‐sustaining sequential processes in which anti‐cancer immune responses lead to the efficient elimination of cancer cells [1]. The cancer–immune cycle has seven steps starting with cancer‐antigen release and ending with cancer cell killing by immune cells. Recently, therapeutics designed to harness the immune system, especially elements of the cancer–immunity cycle, has emerged as a promising treatment for patients with lymphoma. For example, monoclonal antibodies against cytotoxic T‐lymphocyte‐associated antigen 4 (CTLA‐4) and programmed cell death‐1 (PD‐1) molecules are producing long‐term survival of patients with Hodgkin lymphoma and primary mediastinal B‐cell lymphoma [2, 3, 4, 5]. However, the overall response to these immunomodulatory therapeutics in DLBCL is limited [4, 6]; this limitation may be because of the heterogeneity of DLBCL. Indeed, recent genetic landscape studies of DLBCL revealed many recurrently mutated genes targeting the interaction between lymphoma cells and non‐neoplastic cells in the TME, including mutations and aberrant expression of β2 microglobulin protein, a major histocompatibility complex‐I/II molecule, and CD58 [7, 8, 9, 10]. In addition to DLBCL, high‐grade B‐cell lymphoma (HGBL) is considered an immunotherapy‐resistant lymphoma [11]. HGBL with *MYC* and *BCL2* and/or *BCL6* gene rearrangement, mainly derived from germinal center B cells (GCB), was shown to have less T‐cell infiltration and few alterations of inflammation‐related NF‐kB pathway genes [12]. Recently, rare expression of PD‐L1/L2 in HGBL was reported [13, 14]. These observations indicate that the difference between immunotherapy‐sensitive and immunotherapy‐resistant lymphoma depends not only on the intrinsic properties of tumor cells such as genetic alteration of lymphoma cells and cell‐of‐origin (COO), but also whether the TME is ‘immune‐hot’ or ‘immune‐cold’. Furthermore, it appears that converting from immune‐cold to immune‐hot TME is required to respond to an immune checkpoint inhibitor [15]. For example, in patients with DLBCL who achieved a clinical remission, serum levels of PD‐L1 (sPD‐L1) reduced, which was attributed to an immunological impact of therapy [5]. Therefore, it is important to understand the mechanisms of immunosuppression or immune surveillance evasion of DLBCL to select patients for whom immunotherapy is likely to be effective, and to find new therapeutic targets.

## Materials and methods

### Bioinformatics analysis

#### Datasets

Clinical data and gene expression data were obtained from the Gene Expression Omnibus (GEO) database (https://www.ncbi.nlm.nih.gov/geo/). We used four datasets as shown in supplementary material, Table S1. For prognosis analyses, patients treated with rituximab, cyclophosphamide, doxorubicin, and vincristine (R‐CHOP) (469 cases out of 928 cases of GSE117556 [10] dataset and 233 cases out of 414 cases of GSE10846 [16] dataset) were included in this study. For the analyses of clinicopathological features, all patients were included whose clinical data were available (928 cases from GSE117556 and 220 cases from GSE4475 [17] datasets).

#### Statistical analysis

Statistical analyses were performed using R v4.1.1. Values of the array data were robust Z‐scored within a dataset using the ‘sights’ package of R. Two‐ or three‐grade stratification of DLBCL by CD24 expression was performed by k‐means clustering using the ‘arules’ package. Univariate and multivariable analyses of overall survival (OS) and progression‐free survival (PFS) were performed on the R‐CHOP treated cohort (*n* = 469; GSE117556 and *n* = 233; GSE10846) using Cox regression. Hazard ratios with 5% and 95% confidence intervals and *P*‐values were reported for the model covariate. Differences in survival curves were assessed using log‐rank tests or Cox proportional hazard model.

#### Gene set enrichment analysis

The gene expression profile (GEP) of CD24‐high cases versus CD24‐low cases was compared using gene set enrichment analysis (GSEA). The Broad Institute JAVA Desktop software Version 4.03 of GSEA was utilized. Enrichment of gene set signatures was evaluated using the Hallmark Gene Sets Collection version 7.2 with a two‐class analysis, 1,000 permutations of gene sets, and weighted metrics. Gene sets with false discovery rate (FDR) *q*‐value <0.25 or *P*‐value <0.05 were considered significant.

#### Estimation of immune microenvironment cell fractions

CIBERSORT algorithm developed by Newman *et al* estimates cell proportion based on gene expression data [18]. LM22, a leukocyte gene signature, is used to estimate 22 hematopoietic cells with high sensitivity and specificity. Using this algorithm, we estimated the fractions of immune microenvironment cells of DLBCL samples in GSE117556 through the CIBERSORT web portal (https://cibersort.stanford.edu/). Then, we compared the proportion of each immune cell between the CD24‐high and ‐low groups.

### Tissue microarray

Tumor biopsy specimens and clinical data were obtained retrospectively from 190 patients diagnosed with diffuse large B‐cell lymphoma, not otherwise specified (DLBCL, NOS) from 2008 to 2015 at Saitama Medical Center, Saitama Medical University. The institutional ethics committee of Saitama Medical Center, Saitama Medical University, approved the use of all specimens and clinical data collections (No. 1966‐V). All patients were diagnosed according to the World Health Organization classification of hematopoietic and lymphoid tissues, 2017 [19]. Histopathological examination, including immunohistochemistry, was executed using a tissue microarray (TMA). Morphologically, high‐grade lymphoma was excluded from analysis. After three pathologists (NT, SM, and JT) selected two representative areas, the corresponding tissue cores of 2.0 μm diameter were taken from the paraffin blocks and transferred to the recipient block using a tissue microarrayer (Azumaya, Tokyo, Japan). Normal tonsil tissue and liver tissue were included in each TMA block as batch controls for staining.

### Immunohistochemistry and fluorescence *in situ* hybridization

The antibodies used for pathological diagnosis of DLBCL were as follows: CD20 (L26), CD10 (56C6), and BCL6 (LN22) (Leica Biosystems, Wetzlar, Germany); MUM‐1 (MUM1p), BCL2 (clone 124), and Ki‐67 (MIB‐1) (Dako, Glostrup, Denmark); and C‐MYC (Y69) (Abcam, Cambridge, UK). Cases were subclassified into two groups, GCB type and non‐GCB type, using the algorithm described by Hans *et al* [20]. A monoclonal antibody (clone 32D12, BMA Biomedicals, Augst, Switzerland) was used for the detection of CD24. For the detection of pan‐macrophages and M2 macrophages, anti‐CD68 antibody (PG‐M1; Dako) and anti‐CD163 antibody (10D6; Leica Biosystems) were used, respectively. Immunohistochemical (IHC) staining was performed using BOND Polymer Refine Detection in an auto‐immunostainer, BOND‐III (Leica Biosystems). In brief, 3 μm sections of formalin‐fixed, paraffin‐embedded tissues were deparaffinized with xylene and dehydrated in a graded series of alcohol. After heat‐induced antigen retrieval, the sections were incubated with primary antibodies for 15 min at 37°C. Fluorescence *in situ* hybridization was performed on 1 μm thick sections of TMAs using the Vysis LSI dual‐color break‐apart probes for *MYC*, *BCL2*, and *BCL6* (Abbott Molecular, Des Plaines, IL, USA) with thresholds for positivity of 14%, 8%, and 9%, respectively. After image acquisition with a fluorescence microscope (Olympus Corporation, Tokyo, Japan) equipped with appropriate filter sets, images were evaluated using BioView SOLO System (BioView Ltd, Rehovot, Israel).

### Image analysis

The immunostained specimens were quantified using image analysis software, QuPath [21]. In brief, after whole‐slide scanning of the immunostained specimens using Slideview VS200 (Olympus Corporation), the images were opened with QuPath software, dearrayed, and deconvoluted into hematoxylin and DAB images. For CD3‐, CD8‐, CD68‐, and CD163‐stained specimens, ‘positive cell detection’ was performed in QuPath. For CD24‐ and MYC‐stained specimens, H‐scores [22] were calculated for each TMA core based on the extent and intensity of cytoplasmic staining (3 × % of strongly staining cytoplasm + 2 × % of moderately staining cytoplasm + 1 × % of weakly staining cytoplasm, giving a range of 0–300).

## Results

### Immune checkpoint molecules in DLBCL

**Figure 1:**
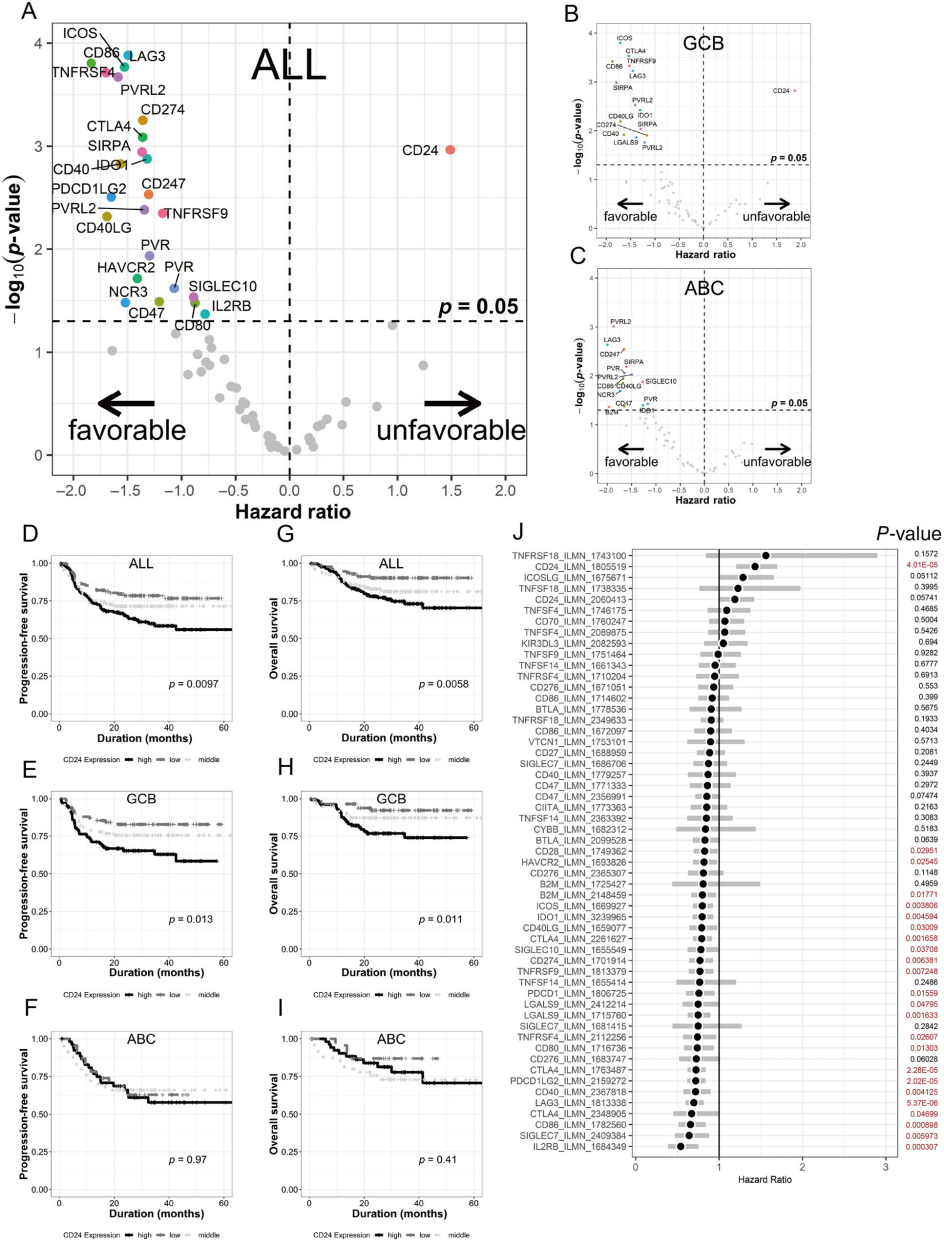
Prognostic impact of immune checkpoint‐related molecules. (A–C) Volcano plots depicting the hazard ratio of OS and *P*‐value of the expressed gene levels in patients with DLBCL treated with R‐CHOP in all cases (A), GCB‐DLBCL (B), and ABC‐DLBCL (C). (D–I) Kaplan–Meier curves showing that CD24 expression is an unfavorable candidate gene in all cases (D, G), GCB cases (E, H), and ABC cases (F, I). *P*‐values were calculated by log‐rank test. (J) Forest plot of hazard ratios. Bars indicate 95% confidence interval. Multivariate analysis using the Cox regression method considering variables IPI and COO indicates that CD24 was the only ‘unfavorable’ gene.

To identify immune‐modulating genes that correlate with the prognosis of patients with DLBCL, we conducted a prognosis analysis that stratified the expression of 43 genes reported as immune checkpoint molecules or their ligands including, *ADORA2A*, *B2M*, *BTLA*, *CD24*, *CD27*, *CD28*, *CD40*, *CD40LG*, *CD47*, *CD70*, *CD80*, *CD86*, *CD247*, *CD274*, *CD276*, *CIITA*, *CTLA4*, *CYBB*, *HAVCR2*, *ICOS*, *ICOSLG*, *IDO1*, *IL2RB*, *KIR3DL3*, *LAG3*, *LGALS9*, *NCR3*, *PDCD1*, *PDCD1LG2*, *PVR*, *PVRL2*, *SIGLEC10*, *SIGLEC7*, *SIRPA*, *TIGIT*, *TNFRSF18*, *TNFRSF4*, *TNFRSF9*, *TNFSF14*, *TNFSF18*, *TNFSF4*, *TNFSF9*, and *VTCN1*. A dataset from the REMoDL‐B trial [10, 23] was obtained from the GEO database (GSE117556). First, we stratified the expression of each gene into three grades, high expression, middle expression, and low expression by k‐means clustering, and then calculated the hazard ratio between the high expression group and low expression group of each gene. The number of cases in the groups and the maximum/minimum value of the robust scores are shown in supplementary material, Table S2. A volcano plot depicting the hazard ratios of OS and *P*‐values of the expressed gene levels in patients with DLBCL treated with R‐CHOP is shown in Figure 1A–C. Most of the molecules were plotted as ‘favorable’, and only CD24 was plotted as having an ‘unfavorable’ outcome (Figure 1A). We conducted the same assay for the GCB (Figure 1B) and activated B‐cell (ABC) (Figure 1C) types of DLBCL. CD24 was ‘unfavorable’ for GCB‐DLBCL (Figure 1B), whereas no molecule was ‘unfavorable’ for ABC‐DLBCL (Figure 1C). Kaplan–Meier curves also showed that the expression of CD24 is an unfavorable candidate gene for all analyzed cases, and for GCB cases, there was no statistical difference in OS and PFS between CD24‐high and CD24‐low groups of ABC type (Figure 1D–I). As international prognostic index (IPI) and COO significantly predict OS [24, 25], multivariable analysis of prognosis showed that CD24 was the only gene associated with a worse OS independent of the IPI and COO of the lymphoma, with *P*‐value less than 0.05 (Figure 1J). We conducted the same analysis with independent datasets of GSE10846 [16] and GSE181063 [9, 26]. CD24 was plotted as ‘unfavorable’ for all subtypes and for the GCB subtype (supplementary material, Figure S1) in these datasets. Kaplan–Meier curves also showed that CD24 expression is an unfavorable candidate (supplementary material, Figure S1).

### Features of CD24‐high DLBCL

**Figure 2:**
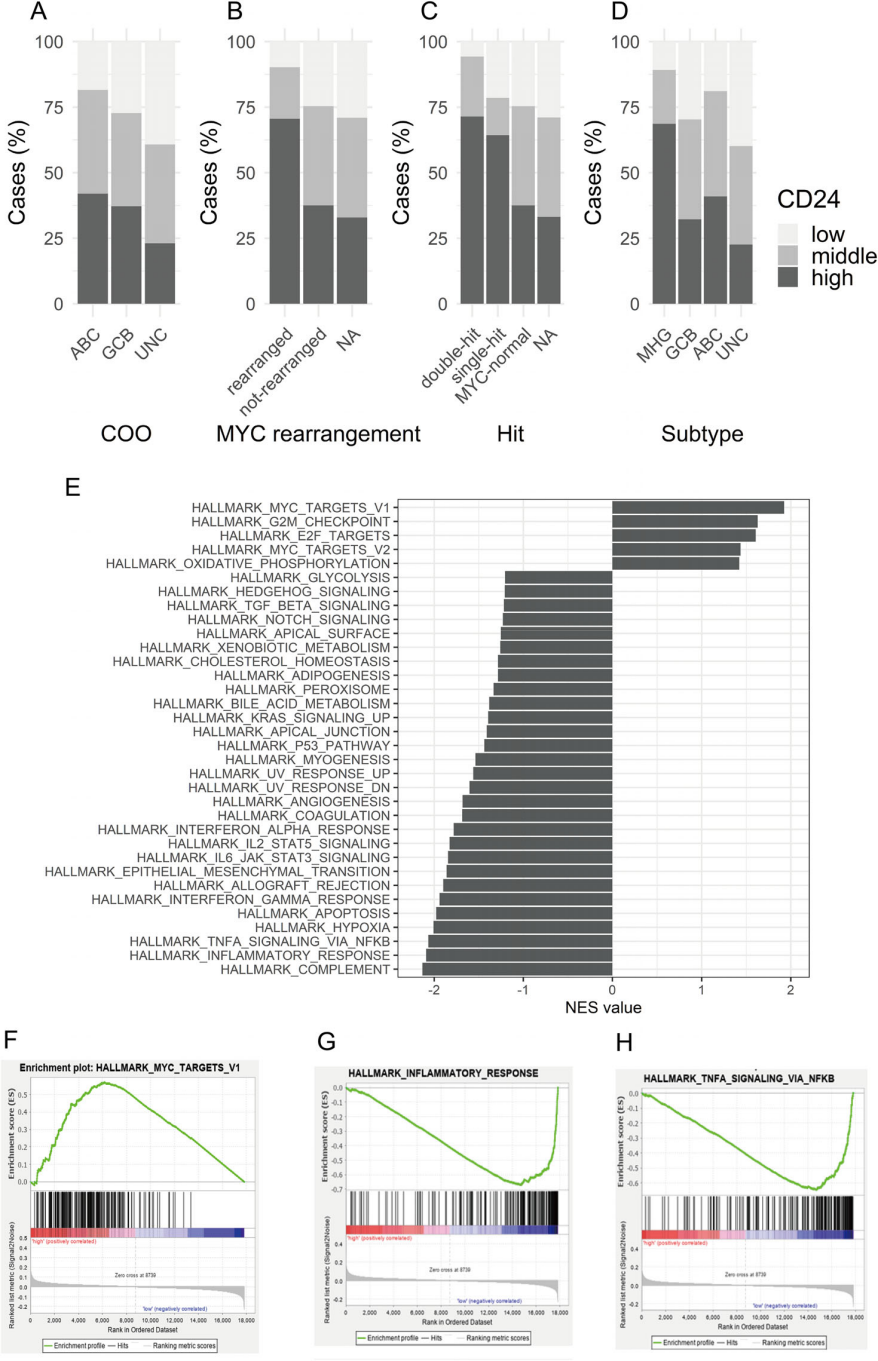
Characteristics of CD24‐high DLBCL. (A) Bar plot indicating that the numbers of CD24‐high and CD24‐low cases were comparable in GCB and ABC DLBCL. UNC, unclassified. (B–D) CD24‐high cases were enriched in *MYC* rearrangement cases (B), single‐ or double‐hit cases (C), and MHG cases (D). NA, not assessed. (E) Bar plot of the GSEA. NES, normalized enrichment score. (F) The most enriched gene set of the CD24‐high group was ‘hallmark_MYC_targets’. (G, H) The top two enriched gene sets in the CD24‐low group were related inflammatory genes, such as ‘Hallmark_inflammatory_response’ and ‘Hallmark_TNFa_signaling_via_NFkB’.

As CD24‐expressing lymphoma had a poor prognosis in all three datasets examined, we next examined the characteristics of CD24‐expressed lymphomas. We explored the clinicopathological features of 928 cases (GSE10846) such as COO, molecular subtypes, and several genetic alterations including translocation of *C‐MYC*, *BCL2*, and *BCL6*. As shown in Figure 2 and supplementary material, Table S3, there was no difference in IPI score, LDH, *BCL2* rearrangement, and *BCL6* rearrangement between CD24‐high and ‐low groups. The cases with translocation of the *MYC* gene were aggregated in the CD24‐high group (Figure 2B, *p* < 0.001). Moreover, the number of double‐hit lymphomas was higher in the CD24‐high group than in the CD24‐low group (Figure 2C, *p* < 0.001). Sha *et al* categorized the cases of the dataset into three subtypes, GCB, ABC, and molecular high‐grade (MHG) lymphoma, according to GEPs [10]. MHG was more frequent in the CD24‐high group than the CD24‐low group (20 and 8.9%, respectively, *p* < 0.001; Figure 2D). Furthermore, comparing GEP of the CD24‐high versus low DLBCL by GSEA showed that several genesets related to MYC targets (Figure 2E,F), G2M checkpoint, and E2F targets were the most enriched in the CD24‐high group compared to CD24‐low cases (Figure 2E,F and supplementary material, Table S4), whereas genesets related to inflammation such as ‘complement’, ‘inflammatory response’, and ‘TNFα signaling via NF‐κB response’ were enriched in the CD24‐low group (Figure 2E,G,H and supplementary material, Table S5).

**Figure 3:**
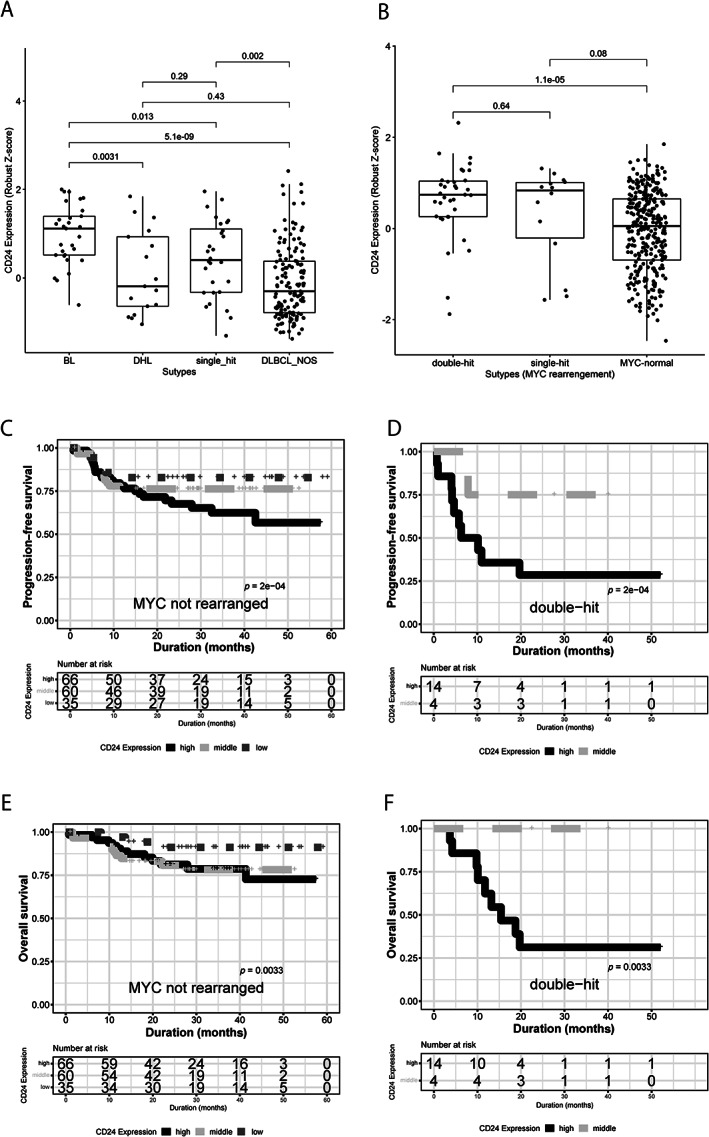
Relationship between *MYC* rearrangement and CD24 expression. (A, B) CD24 expression in BL, double‐hit lymphoma (DHL), single‐hit lymphoma, and DLBCL, NOS (A; Hummel dataset; GSE4475) and double‐hit, single‐hit and MYC‐normal DLBCL (Sha dataset; GSE117556). (C–F) Kaplan–Meier curves show that CD24 expression is an unfavorable candidate gene both in non‐*MYC*‐rearranged DLBCL and in double‐hit lymphoma (Sha dataset; GSE117556). *P*‐values were calculated using a Cox proportional hazard model.

The implication of *MYC* rearrangement in CD24‐expressed lymphoma prompted us to explore the relationship between *MYC* aberration and CD24 expression in B‐cell lymphoma including Burkitt lymphoma (BL). CD24 expression was higher in BL than in DLBCL including double‐hit lymphoma, lymphoma with single hit of the *MYC* gene, and DLBCL, NOS in Hummel's dataset (GSE4475). CD24 expression was also higher in single‐hit lymphoma than in DLBCL, NOS (*p* = 0.002). In Sha *et al*'s dataset (GSE117556), CD24 expression was higher in double‐hit or single‐hit lymphoma than in DLBCL without *MYC* rearrangement (*p* = 1.1e‐05 and 0.08, respectively). We also conducted a survival analysis using Sha *et al*'s dataset (GSE117556). The prognosis was worse in patients with high CD24 expression than in those with low CD24 expression, both in double‐hit lymphoma and in the group without *MYC* rearrangement. Multivariate analyses for prognostic factors affecting the OS of patients with DLBCL including MYC expression and genetic aberration of *MYC* showed that CD24 expression is a candidate prognostic factor (Figure 3, Table 1).

**Table 1:** Prognostic factors affecting the OS of patients with DLBCL (GSE117556).

		Univariate analysis		Multivariate analysis 1	Multivariate analysis 2			Multivariate analysis 3	
Characteristic		HR (95% CI)	*p*	HR (95% CI)	*p*	HR (95% CI)	*p*	HR (95% CI)	*p*
CD24 expression	High versus low	2.81 (1.47–5.53)	0.00176*	3.09 (1.59–6.53)	0.00090*	5.62 (1.62–19.53)	0.00647*	5.59 (1.61–19.53)	0.00664*
IPI	High‐intermediate to high versus low to low‐intermediate	2.06 (1.17–3.53)	0.01284*	2.18 (1.22–3.53)	0.00827*	2.88 (1.24–6.53)	0.01434*	2.85 (1.22–6.53)	0.01583*
COO	GCB versus ABC	0.85 (0.45–1.53)	0.32741	1.04 (0.53–2.53)	0.90797	0.59 (0.23–1.53)	0.27847	0.62 (0.23–1.53)	0.32879
*MYC* expression (RNA)	High versus average	1.68 (0.97–2.53)	0.06553	1.47 (0.80–2.53)	0.20955			1.17 (0.43–3.53)	0.75633
*MYC* rearrangement	Rearranged versus not rearranged	4.61 (2.13–9.53)	0.00010*			3.78 (1.64–8.53)	0.00183*	3.55 (1.41–8.53)	0.00695*

CI, confidence interval; HR, hazard ratio.

* *p* < 0.05.

### Components of the TME

**Figure 4:**
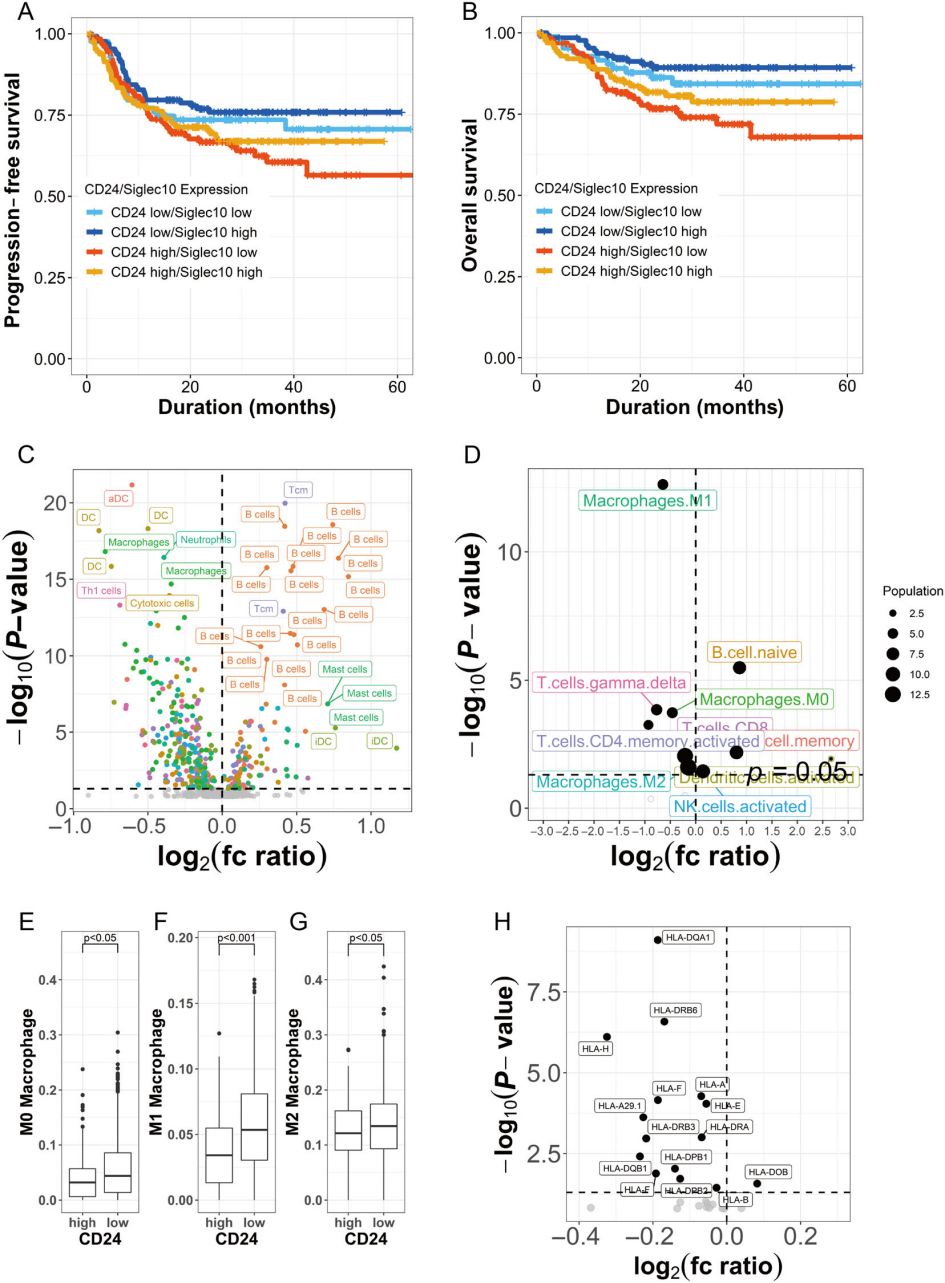
Components of immune microenvironment cells in CD24‐high DLBCL. (A, B) Kaplan–Meier curves showing that the PFS (A) and OS (B) of patients in the CD24‐high/Siglec10‐low group were worse than the other groups. *P*‐values were calculated by log‐rank test. (C) A volcano plot depicts differential expressed cell‐specific genes between CD24‐high and CD24‐low DLBCL. (D) CIBERSORT analysis of the immune microenvironment cells. The population of macrophages in the TME, including M0, M1, and M2, showed a negative correlation with CD24 expression. (E–G) The number of macrophages (M0, M1, and M2) in CD24‐high DLBCL was lower than in CD24‐low DLBCL. (H) A volcano plot showing that expression of most HLA molecules was higher in CD24‐low DLBCL than in CD24‐high DLBCL.

Recently, CD24 was reported as a new ‘don't eat me signal’ which avoids phagocytosis by Siglec10‐expressing macrophages in breast and ovarian cancer, resulting in tumor cell survival [27]. SIGLEC10 is a member of the immunoglobulin superfamily of proteins expressed on the cell surface of macrophages, and functions as a CD24 ligand. To explore the possibility of CD24 as a ‘don't eat me signal’ in DLBCL, we first analyzed the prognosis of DLBCL stratified by CD24 and siglec‐10 expression. The PFS and OS of patients in the CD24‐high/siglec10‐low group were worse than the other groups (Figure 4A,B). Blockade of ‘don't eat me signal’, such as CD47, has been shown to elicit an innate and adaptive immune response *in vitro* and *in vivo* [28, 29]. Therefore, we asked whether CD24 expression on lymphoma cells alters the immune microenvironment to explore immune cell‐specific gene expression in DLBCL (supplementary material, Table S6). A volcano plot depicts the fold change of the cell‐specific genes between CD24‐high and CD24‐low groups (Figure 4C). Most of the B‐cell‐specific genes were plotted in CD24‐high cases, whereas various other immune cell‐specific genes, including macrophage‐specific, dendritic cell‐specific, and T‐cell‐specific genes, were plotted in CD24‐low cases of DLBCL. This result suggested that non‐tumor immune cells are more abundant in CD24‐low DLBCL than in CD24‐high DLBCL. Next, we explored the cell populations in the TME using the CIBERSORT method [18], which allowed us to estimate the populations of component cells from microarray data. The most abundant cells in TME of DLBCL tissue were macrophages, with an average of 13%. The population of macrophages in TME, including M0, M1, and M2, was more abundant in the CD24‐low group (Figure 4D–G). The population of CD4‐positive memory T cells and CD8‐positive T cells also had a negative correlation with CD24 expression (Figure 4D). Moreover, we compared HLA expression between CD24‐high and ‐low groups. Most types of HLA were expressed in CD24‐low DLBCL (Figure 4H). These observations suggest that CD24‐high DLBCL has ‘immune‐cold’ features, and the expression of CD24 on DLBCL cells alters the immune microenvironment of DLBCL.

### CD24 protein expression in DLBCL

**Figure 5:**
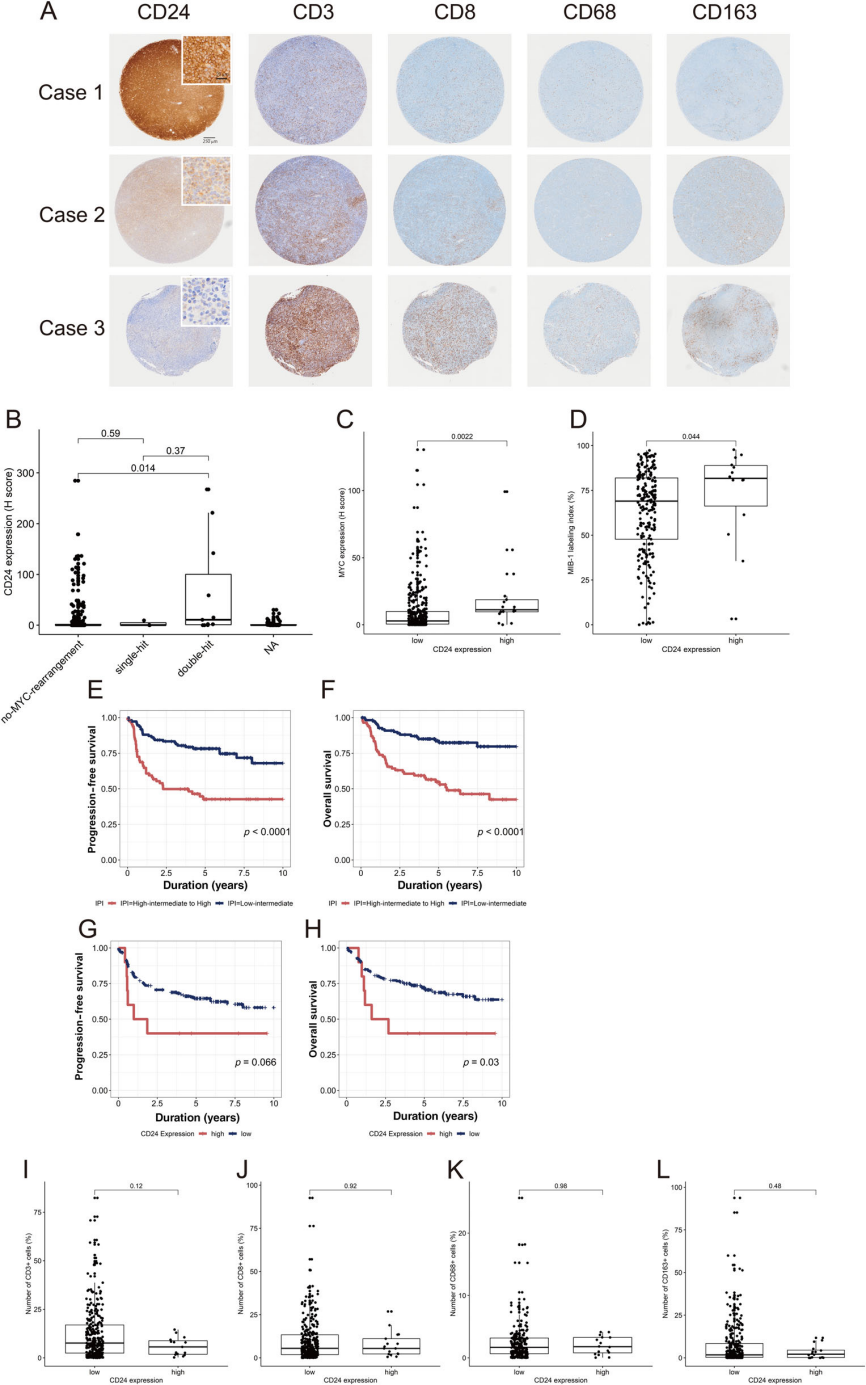
IHC analysis of CD24 expression and macrophages in DLBCL. (A) Representative cases of immunostaining. Cytoplasmic and membranous pattern of CD24 expression in cases 1 and 2, and weak cytoplasmic pattern of CD24 expression in case 3. The numbers of CD3 (+) T cells, CD8 (+) cytotoxic cells, CD68 (+) macrophages, and CD163 (+) M2 macrophages were higher in case 3 than in case 1 or case 2. (B) CD24 expression (H‐score) in non‐*MYC* rearranged, single‐hit, and double‐hit cases. (C) MYC expression (H‐score) in CD24‐low and CD24‐high cases. (D) MIB‐1 labeling index between CD24‐low and CD24‐high cases. (E, F) Kaplan–Meier curves of PFS (E) and OS (F) stratified by IPI. (G, H) Kaplan–Meier curves of PFS (G) and OS (H) stratified by CD24 expression showing that the prognosis of patients in the CD24‐high group was worse than the CD24‐low group. *P*‐values were calculated by log‐rank test. (I–L) Comparison of the number of CD3 (+) T cells (I), CD8 (+) cytotoxic cells (J), CD68 (+) macrophages (K), and CD163 (+) M2 macrophages (L) between CD24‐low and CD24‐high groups.

To validate that CD24 alters the number of immune cells in the TME and influences the prognosis of DLBCL, we performed immunostaining for CD24‐independent series of cases. The summary of cases is shown in Table 2. Representative staining patterns of CD24 are shown in Figure 5; cytoplasmic and membranous pattern (representative in cases 1 and 2), weak cytoplasmic or no staining pattern (representative in case 3). Cases with H‐score of 80 or higher were designated as high and H‐score under 80 as low. As summarized in Table 2, no statistical correlation was found with IPI (*p* = 0.6), stage (*p* = 0.2), soluble IL2 receptor (sIL2R, *p* = 0.8), LDH (*p* = 0.3), and COO (p = 0.7) between CD24‐high and CD24‐low groups. Apart from the results of the analyses of the GEPs, the expression of CD24 did not correlate with the frequency of double‐hit lymphoma (*p* = 0.053) or *MYC* rearrangement (*p* = 0.2). There was however a trend for a higher incidence of double‐hit lymphoma. Protein expression of MYC and MIB‐1 index were higher in the CD24‐high group than in the CD24‐low group (*p* = 0.002 and 0.044, respectively). Although the number of cases analyzed was limited, protein expression of CD24 was associated with poor OS in DLBCL with R‐CHOP/R‐CHOP‐like treatment (Figure 5H, *p* = 0.03). As IPI, MYC expression, and MYC rearrangement also had an impact on prognosis (Table 3), we conducted multivariate analyses. When we performed multivariate analyses 1 and 2, in which MYC expression and MYC rearrangement were included as covariates, respectively, the impact of CD24 expression for OS was marginally significant (*p* = 0.026285 and 0.0433, respectively; Table 3).

**Table 2:** Summary *of* IHC analysis.

	CD24		
Characteristic	High, *N* = 17*	Low, *N* = 318*	*P*‐value^†^
**Age**	70 (66, 73)	70 (60, 77)	0.8
**Gender**			0.5
Female	7 (41%)	155 (49%)	
Male	10 (59%)	163 (51%)	
**PS**			0.6
0	6 (35%)	120 (40%)	
1	5 (29%)	89 (30%)	
2	4 (24%)	32 (11%)	
3	1 (5.9%)	37 (12%)	
4	1 (5.9%)	19 (6.4%)	
**LDH**	224 (204, 368)	271 (212, 430)	0.3
**sIL2R**	1.280 (690, 3.250)	1.320 (654, 3.450)	0.8
**IPI**			0.6
High‐intermediate to high	7 (41%)	140 (47%)	
Low‐intermediate to low	10 (59%)	155 (53%)	
**Stage**			0.2
1	5 (31%)	48 (16%)	
2	4 (25%)	58 (20%)	
3	0 (0%)	47 (16%)	
4	7 (44%)	144 (48%)	
**Rituximab**			0.9
Other	5 (33%)	104 (35%)	
R‐CHOP	10 (67%)	192 (65%)	
**COO**			0.7
GCB	9 (53%)	146 (47%)	
Non‐GCB	8 (47%)	162 (53%)	
**Double_hit**			0.053
Double‐hit	3 (18%)	8 (3.1%)	
No‐*MYC*‐rearrangement	14 (82%)	245 (96%)	
Single‐hit	0 (0%)	3 (1.2%)	
**Recurrence**	10 (62%)	124 (43%)	0.12
**MYC_H‐score**	11 (10, 19)	3 (1, 10)	0.002
**FISH_*MYC***			0.2
Not rearranged	14 (82%)	245 (91%)	
Rearranged	3 (18%)	24 (8.9%)	
**FISH_*BCL2***			0.7
Not rearranged	13 (81%)	202 (83%)	
Rearranged	3 (19%)	42 (17%)	
**FISH_*BCL6***			0.7
Not rearranged	14 (88%)	140 (81%)	
Rearranged	2 (12%)	33 (19%)	
**CD3_PC**	6 (5)	12 (14)	0.12
**CD8_PC**	8 (7)	10 (12)	>0.9
**CD68_PC**	1.97 (1.44)	2.53 (3.17)	>0.9
**CD163_PC**	3 (4)	7 (12)	0.5
**BM_involvement**			0.06
Absent	11 (73%)	235 (90%)	
Present	4 (27%)	25 (9.6%)	
**CNS_involvement**			0.4
Absent	13 (100%)	225 (88%)	
Present	0 (0%)	31 (12%)	

PS; Performance status.

* Median (IQR); *n* (%); mean (SD).

^†^ Wilcoxon rank sum test; Pearson's chi‐squared test; Fisher's exact test.

**Table 3:** Prognostic factors affecting the OS of patients with DLBCL (TMA analysis)

		Univariate analysis		Multivariate analysis 1		Multivariate analysis 2	
Characteristic		HR (95% CI)	*p*	HR (95% CI)	*p*	HR (95% CI)	*p*
CD24 expression	High versus low	2.46 (1.06–5.74)	0.036297*	2.66 (1.12–6.29)	0.026285*	2.50 (1.03–6.06)	0.04333*
IPI	High‐intermediate to high versus low to low‐intermediate	3.64 (2.12–6.27)	0.000003*	3.56 (2.06–6.15)	0.000005*	3.23 (1.82–5.73)	0.00006*
MYC expression	High versus average	2.34 (1.27–4.31)	0.006521*	2.05 (1.10–3.83)	0.024533*		
*MYC rearrangement*	Rearranged versus not rearranged	3.94 (1.97–7.87)	0.000107*			3.36 (1.62–6.93)	0.00107*
MIB1 labeling index	High versus low	1.50 (0.81–2.76)	0.196140				

CI, confidence interval; HR, hazard ratio.

* *p* < 0.05.

We next examined the number of non‐lymphoma TME cells including macrophages and M2 macrophages, CD3‐positive T‐cells, and CD8‐positive cytotoxic cells between CD24‐high and CD24‐low groups. Although there were no statistical differences in the mean number of these non‐tumor immune cells between CD24‐high and CD24‐low groups, only a few TME cells were observed in CD24‐positive DLBCL (Figure 5A,I–L).

## Discussion

Here, we demonstrate that CD24 is a predictor of poor prognosis and a possible immune checkpoint molecule in DLBCL. Several studies have reported that CD24 expression correlates with poor prognosis or metastasis in solid cancers such as ovarian cancer, breast cancer, prostate cancer, and lung cancer [30, 31]. Despite CD24 being used as a differentiation marker of lymphocytes in the hematology field, only a few studies have reported on CD24 in mature B‐cell lymphoma related to its expression and clinical impact. Went *et al* reported that CD24 was expressed in 0–20% of mature B‐cell lymphoma, depending on the subtype of B‐cell lymphoma, and expressed in 9% of DLBCL cases [32]. The frequency of CD24 expression in DLBCL was comparable to our IHC results. Little has been reported on the clinical impact of CD24 in DLBCL [33, 34], and the clinical impact of CD24 is still obscure. As the cases of B‐cell lymphoma in these two reports were diagnosed according to the obsolete Kiel classification or Working Formulation classification, they would include other subtypes in the WHO classification of lymphoma. Qiao *et al* claimed that high CD24 expression correlated favorably with R‐CHOP response and correlated with tumor immunosuppression in ABC‐DLBCL patients [35]. The results of TME status in this report were similar to ours in that CD24‐high cases were immunosuppressive; however, our data showed adverse response to R‐CHOP treatment. This may be due to the difference in the method of CD24 detection and the difference in the setting of the cut‐off value for CD24 expression.

In our analysis of the two independent microarray datasets, CD24‐high DLBCL had a high incidence of *MYC*‐rearrangement and/or high MYC expression. Given that the promoter region of CD24 contains E‐box domains to which MYC can bind, it is conceivable that MYC directly regulates CD24 expression. Indeed, the GSEA of CD24‐high versus CD24‐low DLBCL showed that the most enriched dataset was ‘hallmark_myc_targets’ in CD24‐high DLBCL and MYC expression was higher in CD24‐high DLBCL than in CD24‐low DLBCL by IHC. If MYC regulates CD24 expression directly, CD24 may be one of the surrogate markers of mature B‐cell lymphoma with *MYC* aberrations including HGBL and ‘double expressor’ B‐cell lymphoma. The inclusion of CD24 in the list of HGL signature genes reported by Ennishi *et al* [12] supports this idea. However, the FDR value of the GSEA is relatively high in our analysis, indicating the presence of a complex mechanism of MYC‐related transcription. Indeed, the function of MYC as a transcription factor is also known to be affected by the amount of MYC‐associated molecules such as Max and Mad [36, 37]. The functional relationship between MYC expression and CD24 expression needs to be examined, including *in vitro* experiments. There was no difference in the frequency of CD24‐high cases between GCB/ABC. It is possible that CD24‐high DLBCL forms a group independent of COO, although it tends to be more common in the group with high expression or genetic abnormalities of MYC. In addition to the regulatory mechanism of CD24 expression, the biological features of tumor cells in CD24‐expressing DLBCL should be investigated in further studies.

We showed that the HLA expression was lower in CD24‐high DLBCL than in CD24‐low DLBCL. Several reports, including our previous report, indicated that the loss of HLA expression in DLBCL contributes to escape from immunosurveillance [38, 39, 40]. The results of CIBERSORT analysis suggested that many types of immune cells were more abundant in the CD24‐low group than in the CD24‐high group, suggesting that CD24‐high DLBCL is ‘immune‐cold’, wherein the microenvironmental immune cells are decreased. Scott and Gascoyne classified the TME of B‐cell lymphoma into three patterns: the ‘re‐education pattern’ typified by follicular lymphoma, the ‘recruitment pattern’ typified by Hodgkin lymphoma, and the ‘effacement pattern’ typified by BL [41]. DLBCL is considered to locate between ‘recruitment’ type and ‘effacement’ type. CD24‐high DLBCL seems to be skewed to the ‘effacement’ pattern because immune cells are less than in CD24‐low lymphoma. Several studies have shown that CD24 modulates the immune response in several diseases. For example, genetic alteration of CD24, such as deletion or polymorphisms of the *CD24* gene, is associated with increased risk for autoimmune disease, including systemic lupus erythematosus and multiple sclerosis [42, 43]. Clinical trials have also been conducted to utilize the immunomodulatory capacity of CD24 to prevent the aggravation of Covid‐19 [44, 45] or to suppress the side effects of immunotherapy for solid tumors [46]. Recently, Barkal *et al* reported that CD24 is a new ‘don't eat me’ signal capable of protecting cancer cells from phagocytosis by Siglec‐10 expressing macrophages in breast and ovarian cancer [27]. In our analysis of the dataset, OS and PFS of the Siglec‐10‐low group were inferior to those of the Siglec‐10‐high group of DLBCL. Moreover, the macrophage infiltration was merely observed in CD24‐high DLBCL. This implies that CD24 is also a ‘don't eat me’ signal in DLBCL. CD47 is a well‐known ‘don't eat me’ signal in DLBCL and the therapeutic utility of blocking antibody alone or a combination of blocking antibodies of CD47 ligand, SIRPα, or combination CD47 antibody and rituximab are in clinical trials [47, 48, 49]. As the expression of CD47 and CD24 were not correlated in our analysis, CD24 may be a distinct ‘don't eat me’ signal from CD47, suggesting another possible target of immunotherapy.

Some limitations should be noted. First, the number of cases in the IHC study was relatively small. We could not observe the difference in the frequency of *MYC* aberration between CD24‐high cases and CD24‐low cases in IHC analysis, which is different from the array data. The frequency of *MYC* rearrangement in our hospital data was 9.4% (27/286 cases) and relatively lower than previously reported [17, 50, 51]. Thus, further analysis with a larger sample size is needed. Despite the small number of cases, the prognosis of CD24‐high cases was inferior to CD24‐low cases, suggesting the presence of other factors that affect the prognosis besides *MYC* abnormalities. Second, we could not explore the relationship between mRNA expression and protein expression. The frequency of CD24‐high cases was different between the microarray assay and IHC assay when the cut‐off value in the array experiment was set by k‐means clustering. Comparison between the mRNA and the protein expression of CD24 in the same sample is needed to analyze CD24 expression accurately. Another limitation is that the details of TME constituent cells are still unclear in the TMA analysis. More extensive and detailed analysis of T‐cell subsets, the polarization of macrophages such as M1 and M2, and dendritic cells that function as antigen‐presenting cells will be needed in future studies.

In conclusion, we have shown that the number of immune cells in CD24‐high large B‐cell lymphoma was lower than in CD24‐low cases, suggesting that CD24 on lymphoma cells contributes to escape from immune surveillance as an immune checkpoint signal in DLBCL, leading us to speculate that CD24 will be a new target of immunotherapy of aggressive large B‐cell lymphoma.

## Author contributions statement

MH, SM, NT, TY, JK and JT performed research and analyzed the data. YT, TA, MT and MK collected clinical data. MH wrote the initial draft of the manuscript. All authors contributed to the modification of the draft and approved the final submission.

## Supporting information


**Figure S1.** Prognostic impact of immune checkpoint‐related molecules in the GSE10846 and GSE181063 datasets

**Table S1.** Datasets of gene expression obtained from GEO
**Table S2.** CD24 expression in the GSE117556 dataset
**Table S3.** Clinicopathological features of the GSE117556 dataset
**Table S4.** Top enriched hallmarks in CD24‐high cases by GSEA
**Table S5.** Top enriched hallmarks in CD24‐low cases by GSEA
**Table S6.** Immune cell‐specific genes
