# Supplementary material for: CD24 is a surrogate for ‘immune‐cold’ phenotype in aggressive large B‐cell lymphoma
Source: J Pathol Clin Res. 2022 Mar 14;8(4):340–54. doi: 10.1002/cjp2.266 (PMC9161324; doi:10.1002/cjp2.266)
Supplement: Supplementary file 1 — Figure S1. Prognostic impact of immune checkpoint‐related molecules in the GSE10846 and GSE181063 datasets [file CJP2-8-340-s002.pdf]

CD24 is a surrogate for 'immune-cold' phenotype in aggressive large B-cell lymphoma

M Higashi *et al*. *J Pathol Clin Res* DOI: 10.1002.cjp2.266

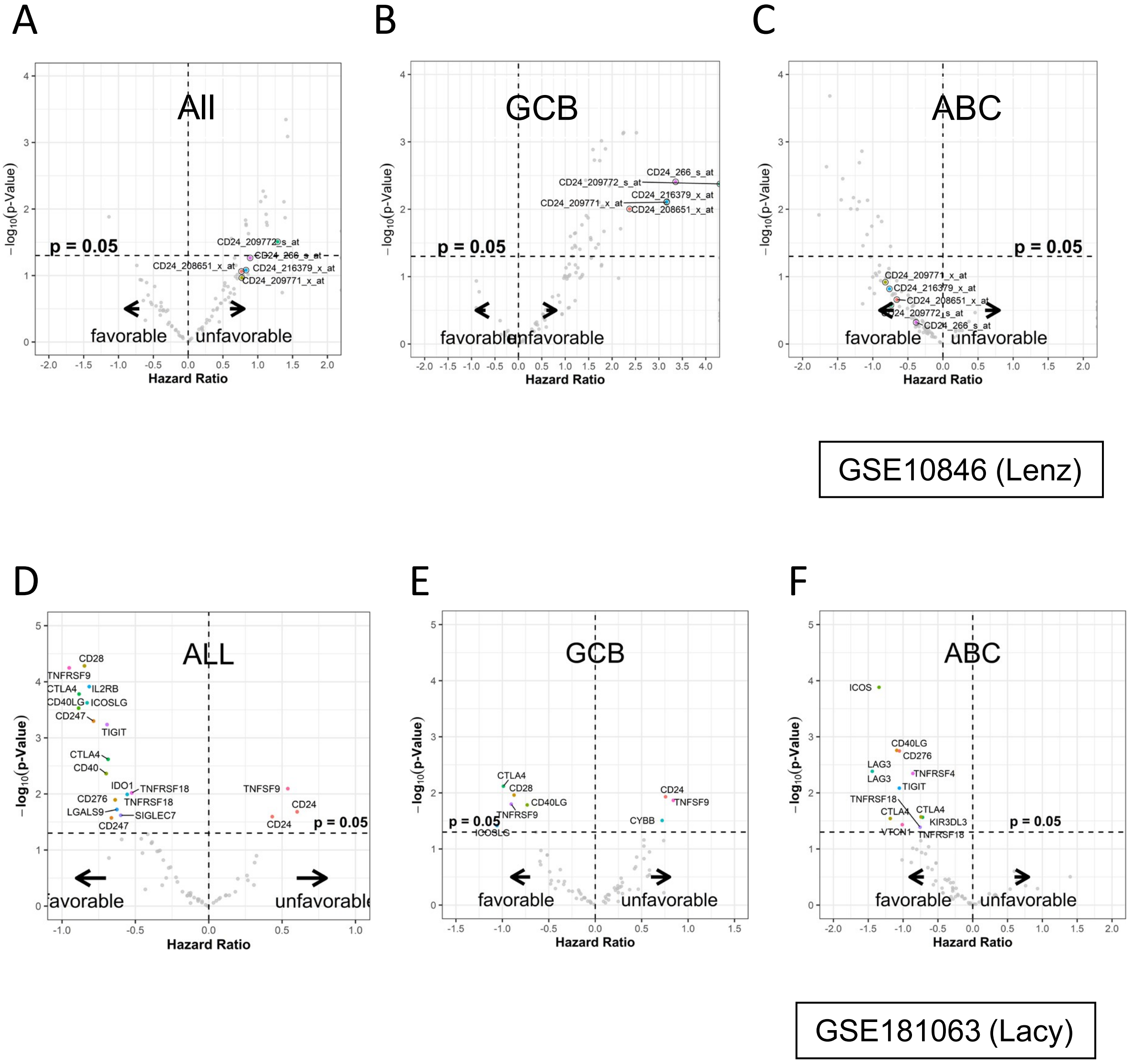

Figure S1. Prognostic impact of immune-checkpoint-related molecules in the GSE10846 and GSE181063 datasets. (A-C) GSE10846 (Lenz). Volcano plots depicting the hazard ratios of overall survival (OS). One of the 5 probesets corresponding to CD24 was plotted "unfavorable" in all subtypes and 4 probesets in all patients or the patients with GCB subtype. (D-F) GSE181063 (Lacy). Volcano plots depicting the hazard ratio of overall survival (OS). Two probesets corresponding to CD24 were plotted "unfavorable" in all subtypes in all patients or the patients with GCB subtype.
